# Comprehensive Analysis of GPSM2: From Pan‐Cancer Analysis to Experimental Validation

**DOI:** 10.1111/jcmm.70527

**Published:** 2025-04-10

**Authors:** Chunjiao Yang, Yuzhe Zhang, Lirong Yan, Aoran Liu, Fang Li, Yanke Li, Ye Zhang

**Affiliations:** ^1^ Department of Oncology The Fifth Affiliated Hospital of Guangxi Medical University & The First People's Hospital of Nanning Nanning China; ^2^ The First Laboratory of Cancer Institute The First Hospital of China Medical University Shenyang China; ^3^ Department of Anorectal Surgery The First Hospital of China Medical University Shenyang China

**Keywords:** biomarker, cell cycle, GPSM2, pan‐cancer, prognosis

## Abstract

G‐protein signalling modulator 2 (GPSM2) plays an important role in maintaining cell polarisation and regulating the cell cycle; however, a systematic and comprehensive analysis of GPSM2 in cancer is still lacking. Using extensive multi‐omics data, we explored the pan‐cancer expression levels of GPSM2 from multiple perspectives and its association with prognosis, diagnosis, tumour stemness, immune‐related genes, immune cell infiltration, genomic instability, and response to immunotherapy. We also elucidated the potential pan‐cancer biological functions of GPSM2 using gene set enrichment analysis (GSEA) and searched for targeted drugs that affect GPSM2 expression using connectivity map analysis. To elucidate the effect of GPSM2 on colon cancer, we evaluated its effect on the biological behaviour of two colon cancer cell lines. In this study, GPSM2 was systematically analysed and shown to have satisfactory performance in disease diagnosis and prognostic prediction of various cancers. G‐protein signalling modulator 2 plays an important role in the genesis and development of various tumours and is a potential tumour diagnostic and prognostic biomarker as well as an anti‐cancer therapeutic target.

**Abbreviations**
BLCA: bladder urothelial carcinoma
BP: biological processes
BRCA: breast cancer
CC: cellular components
CESC: cervical squamous cell carcinoma
CHOL: cholangiocarcinoma
cmap: connectivity map
COAD: colon adenocarcinoma
DFI: disease‐free interval
DFS: disease‐free survival
DLBC: lymphoid neoplasm diffuse large B‐cell lymphoma
DLBCL: diffuse large B cell lymphoma
DNAss: DNA methylation
DSS: disease‐specific survival
EAC: oesophageal adenocarcinoma
ESCA: oesophageal carcinoma
GBMLGG: glioblastoma and low‐grade glioma
GO: Gene Ontology
GPSM2: G‐protein signalling modulator 2
GSEA: gene set enrichment analysis
HNSC: head and neck squamous cell carcinoma
HPA: the Human Protein Atlas
HR: hazard ratio
KICH: kidney chromophobe
KIPAN: pan‐kidney cohort
KIRC: kidney renal clear cell carcinoma
KIRP: kidney renal papillary cell carcinoma
LAML: acute myeloid leukaemia
LGG: brain lower‐grade glioma
LIHC: liver hepatocellular carcinoma
LUAD: lung adenocarcinoma
LUSC: lung squamous cell carcinoma
m1A: 1‐methyladenosine
m5C: 5‐methylcytidine
m6A: N6‐methyladenosine
MESO: mesothelioma
MF: molecular functions
MSI: microsatellite instability
NEO: neoantigen
OS: overall survival
PA: pulmonary adenocarcinoma
PAAD: pancreatic adenocarcinoma
PCPG: pheochromocytoma and paraganglioma
PFI: progression‐free interval
PRAD: prostate adenocarcinoma
READ: rectum adenocarcinoma
RNAss: mRNA expression
SARC: sarcoma
SKCM: skin cutaneous melanoma
STAD: stomach adenocarcinoma
TCGA: The Cancer Genome Atlas
TGCT: testicular germ cell tumours
THCA: thyroid carcinoma
THYM: thymoma
TMB: tumour mutation burden
UCEC: uterine corpus endometrial carcinoma
UVM: uveal melanoma

## 1 Introduction

Cancer has become the leading cause of death in many countries around the world [1]. One of the major causes of high cancer‐related mortality is the lack of early screening markers for cancer and the lack of effective treatment options [2]. Therefore, the identification of clinically relevant biomarkers is critical for early cancer screening and selection of therapeutic modalities [1, 3, 4, 5, 6].

Pan‐cancer analysis can lead to new discoveries in early cancer screening, predicting survival, and developing new targeted therapies [7, 8, 9]. It can reveal the key role of specific genes in the development of certain cancer types as well as the similarities and differences in key biological processes between different tumour types [10, 11, 12]. This can provide important clues for further mechanistic studies while promoting the discovery of additional therapeutic targets [13]. The importance of pan‐cancer analyses has been increasingly recognised by researchers in recent years. It is becoming increasingly important to utilize multi‐omics data to explore disease progression.

G‐protein signalling modulator 2 (GPSM2) regulates the activation of G proteins and reception of extracellular signals. It is involved in cell division directed toward the mitotic spindle and plays an important role in the maintenance of cell polarity and regulation of the cell cycle. G‐protein signalling modulator 2 deficiency leads to the disruption of cell polarity, which in turn causes a series of diseases, such as brain developmental defects [14]. GPSM2 has been demonstrated to play a role in several types of cancers; for example, GPSM2 is involved in the epithelial‐mesenchymal transition process in non‐small cell lung cancer, and it also affects the sensitivity of breast cancer cells to paclitaxel [15, 16]. However, with the rapid development of sequencing technology, no study has been conducted to elucidate the role of GPSM2 in pan‐cancer using available advanced databases and multi‐omics data.

This study aimed to conduct a comprehensive analysis of GPSM2 using extensive multi‐omics data. We examined its pan‐cancer expression, prognostic, and diagnostic value and its relationships with tumour stemness, immune‐related genes, tumour mutation burden, microsatellite instability, immune neoantigens, immune scores, and immune cells. Additionally, it is crucial to analyse the potential mechanism of action of GPSM2 in various cancers and identify targeted therapies. Finally, we conducted in vitro experiments using two colon cancer cell lines to confirm the effects of GPSM2 on biological behaviour. Our objective was to explore the potential of GPSM2 as a novel biomarker and therapeutic target for cancer diagnosis and prognosis. We also sought to improve our understanding of the involvement of GPSM2 in cancer progression and treatment, while providing critical insights for future mechanistic studies.

## 2 Material Methods

### 2.1 Data Collection and Processing

Data for this study were collected and processed using The Cancer Genome Atlas (TCGA, https://portal.gdc.cancer.gov/) and the University of California, Santa Cruz (UCSC, https://xena.ucsc.edu/). Importantly, ethical review and patient informed consent were not required, as the study adhered to the established guidelines of TCGA and UCSC.

### 2.2 Pathologic Images of GPSM2 via Human Protein Atlas Database

We obtained immunohistochemical images of GPSM2 associated with tumour and normal tissues from the Human Protein Atlas database (HPA, https://www.proteinatlas.org/) [17].

### 2.3 Prognostic Analysis of Pan‐Cancer

We investigated several critical survival metrics, including overall survival (OS), disease‐specific survival (DSS), disease‐free interval (DFI), and progression‐free interval (PFI). We assessed the effect of GPSM2 on specific prognostic types of each malignancy using Cox regression and lagrank methods, and we subsequently presented the findings as heat maps.

### 2.4 Tumour Stemness, Staging Expression, and Immune‐Related Analyses

We performed correlation analysis using SangerBox 3.0 (http://sangerbox.com) [18]. We extracted the expression data of the GPSM2 gene from each sample. We then calculated the Pearson correlation coefficient between the gene and immune infiltration scores for each tumour using the corr.test function from the R package psych (version 2.1.6) to find significant correlations. Differences in the expression of genes in each tumour in samples with different clinical stages were calculated. Pearson's correlation was also calculated for GPSM2 and marker genes for Inhibitory and Stimulatory. TMB, MSI score, and Neoantigen data were calculated for each tumour using the tmb function of R package maftools (version 2.8.05). The immune infiltration score for each patient in each tumour was reassessed based on gene expression using the R package IOBR's deconvo_ips method [19, 20]. Tumour stemness scores were obtained from previous studies and their Pearson correlation in each tumour was calculated [21]. In addition, we used the ssGSEA method to calculate scores for 15 immune‐related pathways and finally calculated the correlation of all scores with GPSM2.

### 2.5 Spatial Transcriptome Analysis

Spatial transcriptome data for OV, LUSC, LUAD, and BRCA were obtained from GSE203612‐GSM6177614, LUNG_FFPE_CytAssist_10x (https://www.10xgenomics.com/), BrainMetastasis_GSE179572‐GSM5420754, and GSE203612‐GSM6177603_NYU_BRCA2. We utilised the Sparkle database (https://grswsci.top) for analysis [22, 23]. Relevant studies on the sources of single‐cell transcriptome data were referenced to ensure the scientific validity and accuracy of the screening criteria. Subsequently, a signature score matrix was constructed by calculating the average expression of the top 25 specifically expressed genes of various cell types in the scRNA‐seq reference for each locus. Finally, using the get_enrichment_matrix and enrichment_analysis functions in the Cottrazm software package, we successfully generated an enrichment scoring matrix, which provided strong support for subsequent cellular composition analysis. The cell type with the highest content in each microregion was calculated, and the SpatialDimPlot function in the Seurat package was used to visualise the maximum cellular composition of each microregion. The SpatialFeaturePlot function in the Seurat package was used to visualise the gene expression landscape in each microregion. Correlation between cell content and cell content, and between cell content and gene expression in all spots were calculated using Spearman correlation analysis and visualised using the linkET package.

### 2.6 Single‐Cell Analysis and Drug Analysis

Single‐cell data were obtained from Tumour Immune Single‐cell Hub 2 (http://tisch.comp‐genomics.org/home/). Information from the GDSC (Genomics of Drug Sensitivity in Cancer) database, PRISM database, and Cancer Therapeutics Response Portal (CTRP) database were utilised. Analysed using Sparkle database. All cancer types were selected, and heatmaps were used to demonstrate the expression levels of GPSM2 in single cells. In addition, Spearman's correlation between GPSM2 and drug IC50 or AUC values was calculated.

### 2.7 Differential Expression Analysis and Set Enrichment Analysis

We conducted a differential expression analysis of GPSM2 using the DESeq R package. Next, we performed gene set enrichment analysis (GSEA) with the clusterProfiler R package using the results from the differential expression analysis. We obtained the 200 GPSM2‐associated genes that exhibited the most similar expression patterns to GPSM2 across various cancer types from the GEPIA2 database. To further explore the potential functions of GPSM2, we conducted Gene Ontology (GO) analysis on GPSM2‐related genes, focusing on biological processes (BP), cellular components (CC), and molecular functions (MF), along with KEGG analysis.

### 2.8 Connectivity Map (Cmap) Analysis

The differential expression analysis of high versus low gpsm2 levels identified genes that may be regulated across various cancer types. Comparing gene‐related features with cMAP gene features resulted in similarity scores for 1288 compounds. Compounds that received lower similarity scores may inhibit pro‐cancer effects mediated by genes.

### 2.9 Cell Culture

The COAD cells (RKO and SW480) were purchased from the Shanghai Cell Bank of the Chinese Academy of Sciences. Cells were maintained in RPMI‐1640 medium supplemented with 10% fetal bovine serum and grown at a carbon dioxide concentration of 5% and a temperature of 37°C.

### 2.10 Cell Cycle Analysis, Apoptosis Assay, Transwell Assay

The collected cells were washed with pre‐cooled PBS and fixed overnight with 70% cold ethanol at 4°C. The next day, the cells were stained with a staining solution (Thermo Fisher, F10797) at room temperature (30 min). The cells were then inoculated on a 6‐well plate and stained in the dark with Annexin‐V/PI according to the manufacturer's instructions (Beyotime, C1062M). The data were detected by flow cytometry and visualised using FlowJo‐V10 software.

The cells were inoculated into the upper lumen of transwell at a density of 1 × 10^4^ cells/mL (Corning; 3422, United States). According to the proportion of serum‐free medium: Matrigel gel = 7:1. After dilution, 100ul of diluted Matrigel matrix gel was dropped on the upper surface of the chamber. The lower cavity was placed in 500 μL medium containing 10% FBS. After 24 h of incubation, the cells were swabbed onto the upper surface. The films were fixed in methanol for 30 min and stained with crystal violet for 15 min. The cells that passed through the membrane were observed under a microscope and photographed.

### 2.11 Western Blot

Use RIPA lysis buffer to extract the proteins. Determine the protein concentration using the BCA method. Proteins are separated using sodium lauryl sulfate‐polyacrylamide gel electrophoresis and then transferred to a polyvinylidene fluoride membrane following standard protocols. First, block the membrane with milk powder for 1 h, then incubate it overnight with the primary antibody at 4°C. The next day, incubate the secondary antibody for 1 h at room temperature. Use enhanced chemiluminescence kits to identify the protein bands, and analyse the results using ImageJ software. The antibody used was GPSM2 (Proteintech, 26798‐1‐AP).

### 2.12 Statistical Analysis

The pearson rank test was employed to assess the correlation between the two groups. A p‐value of less than 0.05 was deemed statistically significant, with significance levels indicated as follows: *p < 0.05, **p < 0.01 and ***p < 0.001. Results were analysed both visually and statistically using GraphPad Prism 8.0 (GraphPad Software LLC, San Diego, USA) and R software (version 4.0.2). All data were independently repeated at least three times and presented as mean ± standard deviation. A *p* < 0.05 was considered statistically significant.

## 3 Results

### 3.1 Pan‐Cancer Expression of GPSM2

**FIGURE 1:**
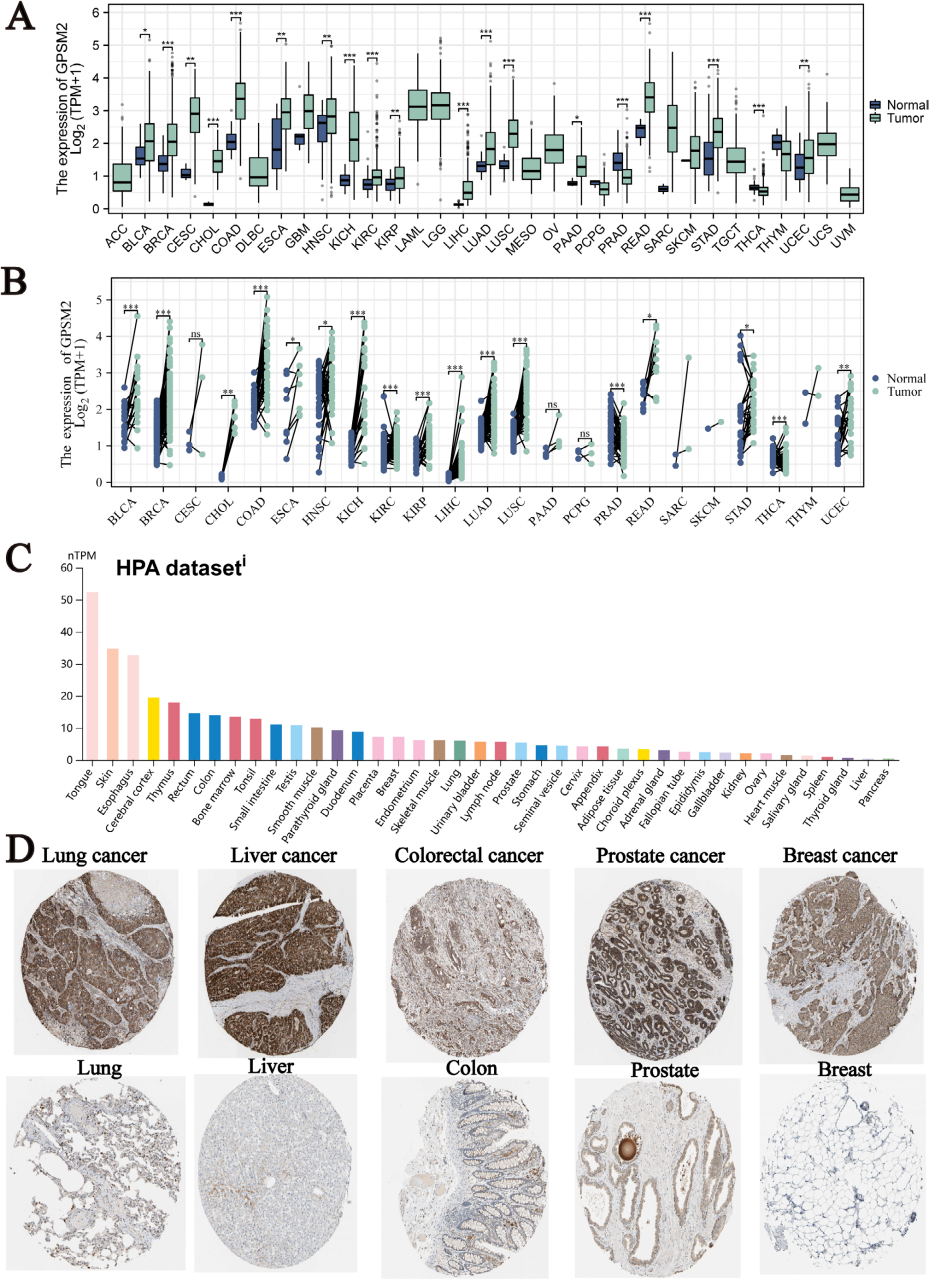
Multi‐omics expression profiling of GPSM2 in pan‐cancer tissues.

Figure 1A shows the expression levels of GPSM2 in various cancers, revealing that GPSM2 is differentially expressed in most tumours. Next, we analysed GPSM2 expression levels in paired samples from The Cancer Genome Atlas. Our analysis yielded similar results (Figure 1B). Data from the Human Protein Atlas show the expression levels of GPSM2 protein in normal human tissues, as illustrated in Figure 1C. We extracted representative immunohistochemical images of the five types of cancer and normal tissues (Figure 1C) that demonstrate the protein expression levels of GPSM2.

### 3.2 GPSM2 Expression in Pan‐Cancer Single Cell Data

**FIGURE 2:**
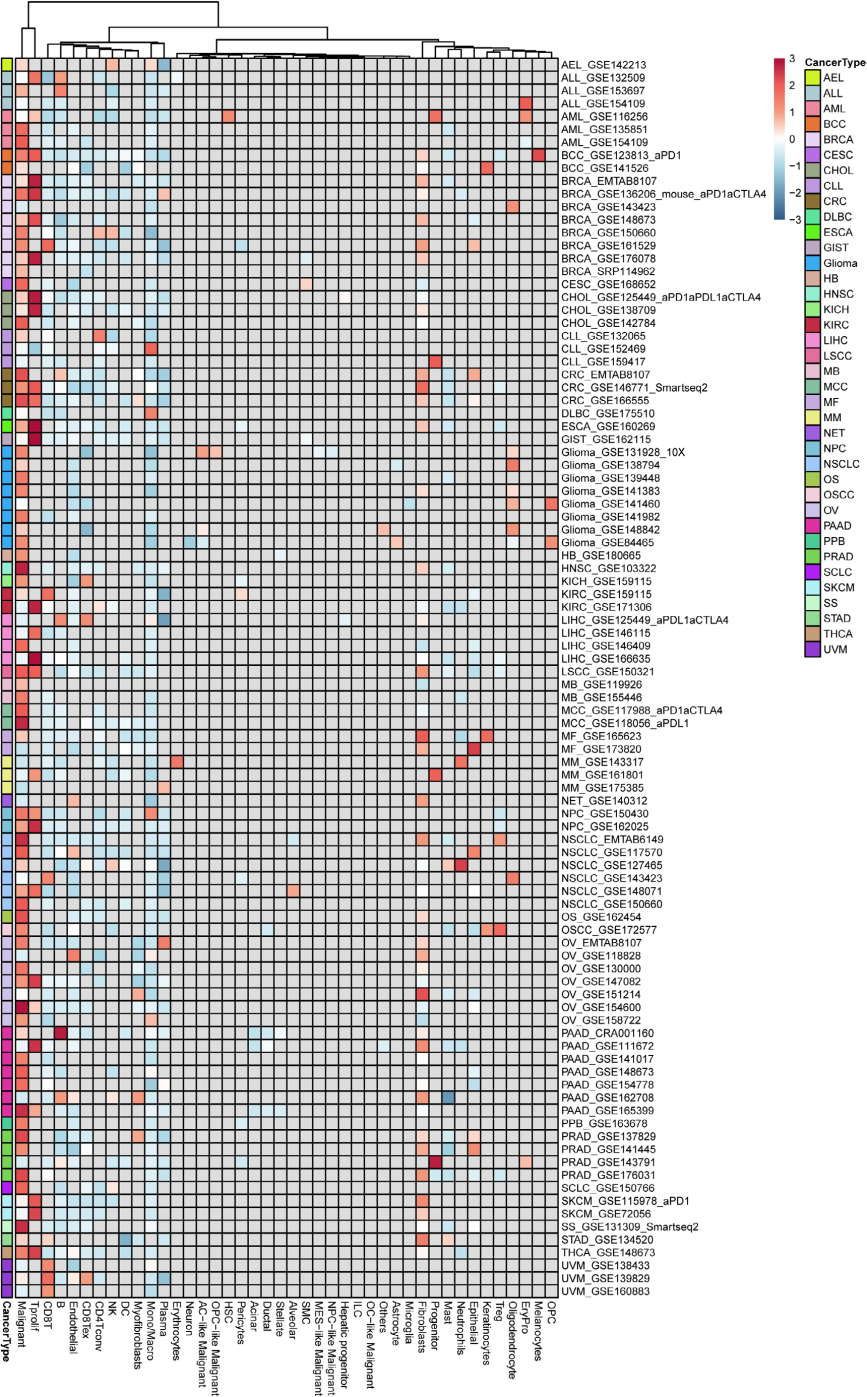
Single‐cell transcriptome reveals GPSM2 expression heterogeneity across cancer species.

By utilising single‐cell data, we gain insights into the primary cell types expressing GPSM2 in the tumour microenvironment. Figure 2 depicts the expression levels of GPSM2 in nearly 100 single‐cell datasets from different tumour types. The results show that GPSM2 is mainly expressed in cancer cells and T proliferating cells.

### 3.3 Relationship Between GPSM2 Expression and Staging

**FIGURE 3:**
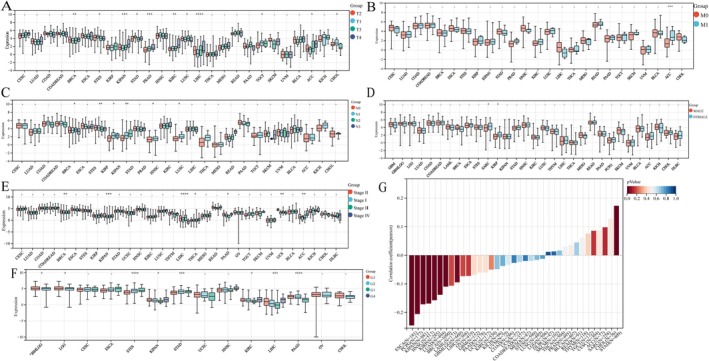
GPSM2 expression levels correlate with clinicopathologic parameters.

To understand the role of GPSM2 expression in cancer development, we evaluated its levels across various cancer types, considering T‐, N‐, and M‐stages, sex, stage, and grading (Figure 3A–F). Additionally, we examined the correlation between GPSM2 expression and age across all cancer types to clarify their relationship (Figure 3G). The results indicated that GPSM2 expression was closely correlated with the clinicopathological stage in thymoma (THYM), pan‐kidney cohort (KIPAN), adrenocortical carcinoma (ACC), and breast invasive carcinoma (BRCA). We found a correlation between GPSM2 expression and patient age in stomach adenocarcinoma (STAD), oesophageal carcinoma (ESCA), pheochromocytoma, and paraganglioma (PCPG).

### 3.4 Pan‐Cancer Prognostic Analysis of GPSM2

**FIGURE 4:**
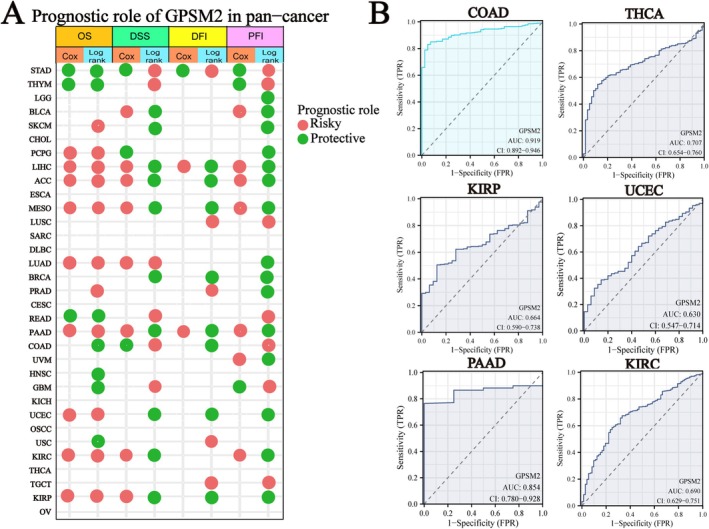
Prognostic and diagnostic value of GPSM2.

Further analyses were conducted to evaluate the pan‐cancer prognostic value of GPSM2. We examined the relationship between GPSM2 expression and the pan‐cancer prognosis. High or low GPSM2 expression was associated with the prognosis of most tumours (Figure 4A; the heatmap shows only *p* < 0.05). A hazard ratio greater than 1 indicated a risk factor, whereas a hazard ratio of less than 1 indicated a protective factor. For instance, high GPSM2 expression is associated with a poor prognosis in patients with lung adenocarcinoma (LUAD). In addition, the diagnostic value of GPSM2 was evaluated using the ROC curve, which revealed that GPSM2 was highly effective in diagnosing colon adenocarcinoma (COAD) (Figure 4B).

### 3.5 Association of GPSM2 With Tumour Stemness and RNA Modification Genes

**FIGURE 5:**
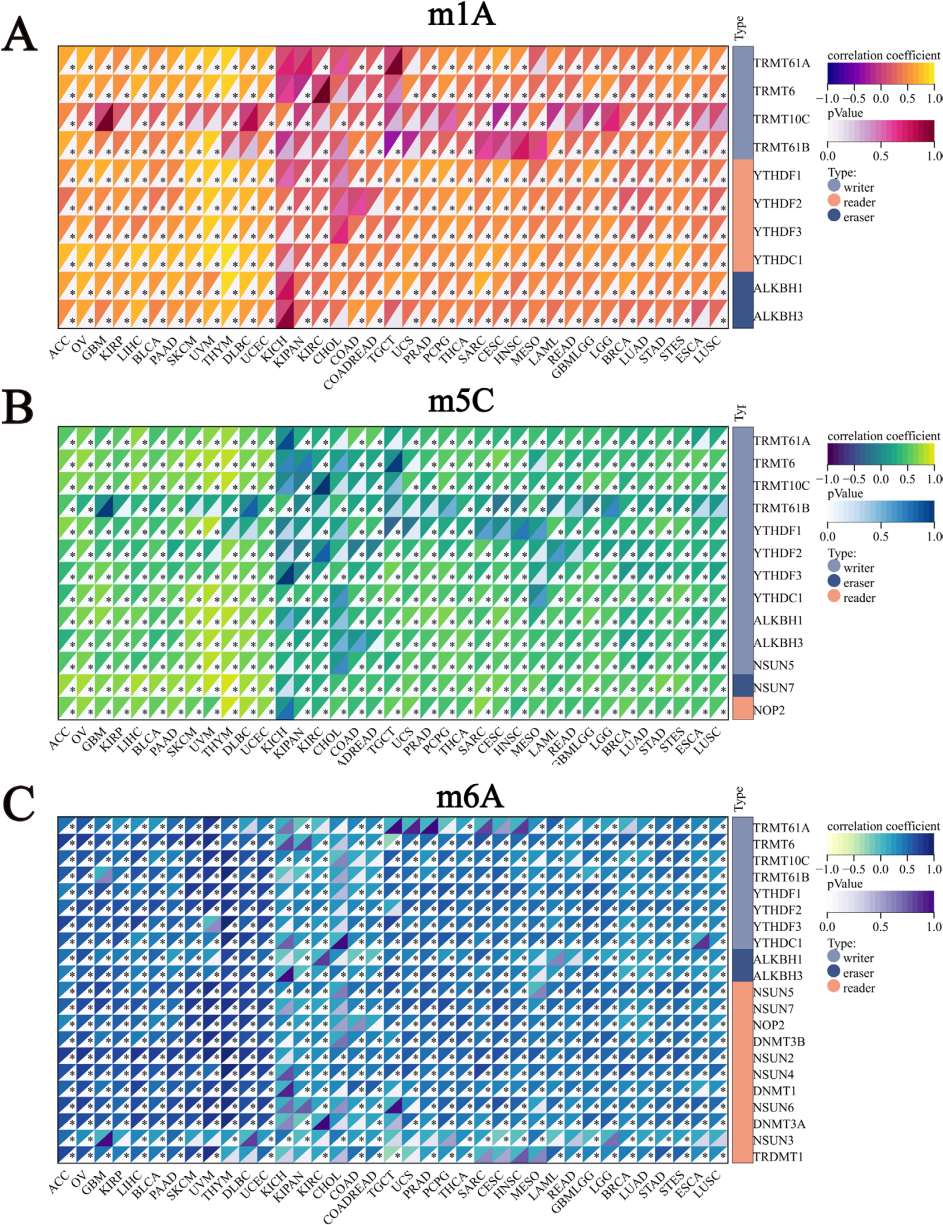
Pan‐cancer co‐expression network of GPSM2 and RNA epitope modification genes.

Modification of mRNA is an important component of epigenetics. Among these, 1‐methyladenosine (m1A), 5‐methylcytidine (m5C), and N6‐methyladenosine (m6A) are the most intensively studied mRNA modifications. We explored the relationship between GPSM2 and the RNA modification genes. G‐protein signalling modulator 2 expression positively correlated with most m1A, m5C, and m6A genes in pan‐cancer tissues (Figure 5A–C).

**FIGURE 6:**
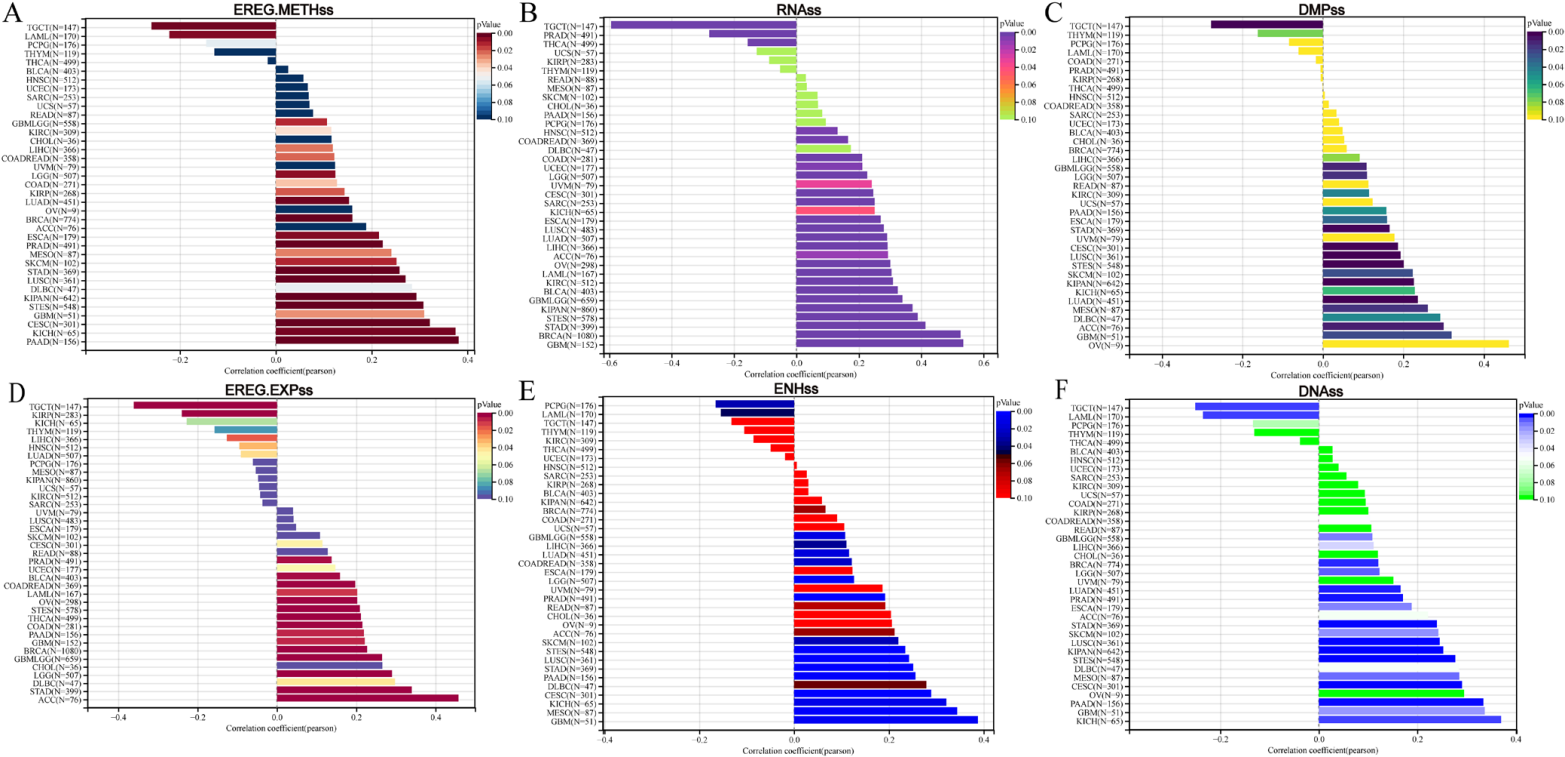
Correlation between GPSM2 expression and tumour stemness characteristics.

In general, the higher the tumour stemness score, the less differentiated the tumour was. We investigated the relationship between GPSM2 expression and tumour stemness scores in six different pan‐cancer dimensions. We found a significant positive correlation between GPSM2 expression and tumour stemness scores in most types of malignant tumours, especially glioblastoma multiforme (GBM), ACC, and STAD (Figure 6A–F).

### 3.6 Role of GPSM2 in Immunity

**FIGURE 7:**
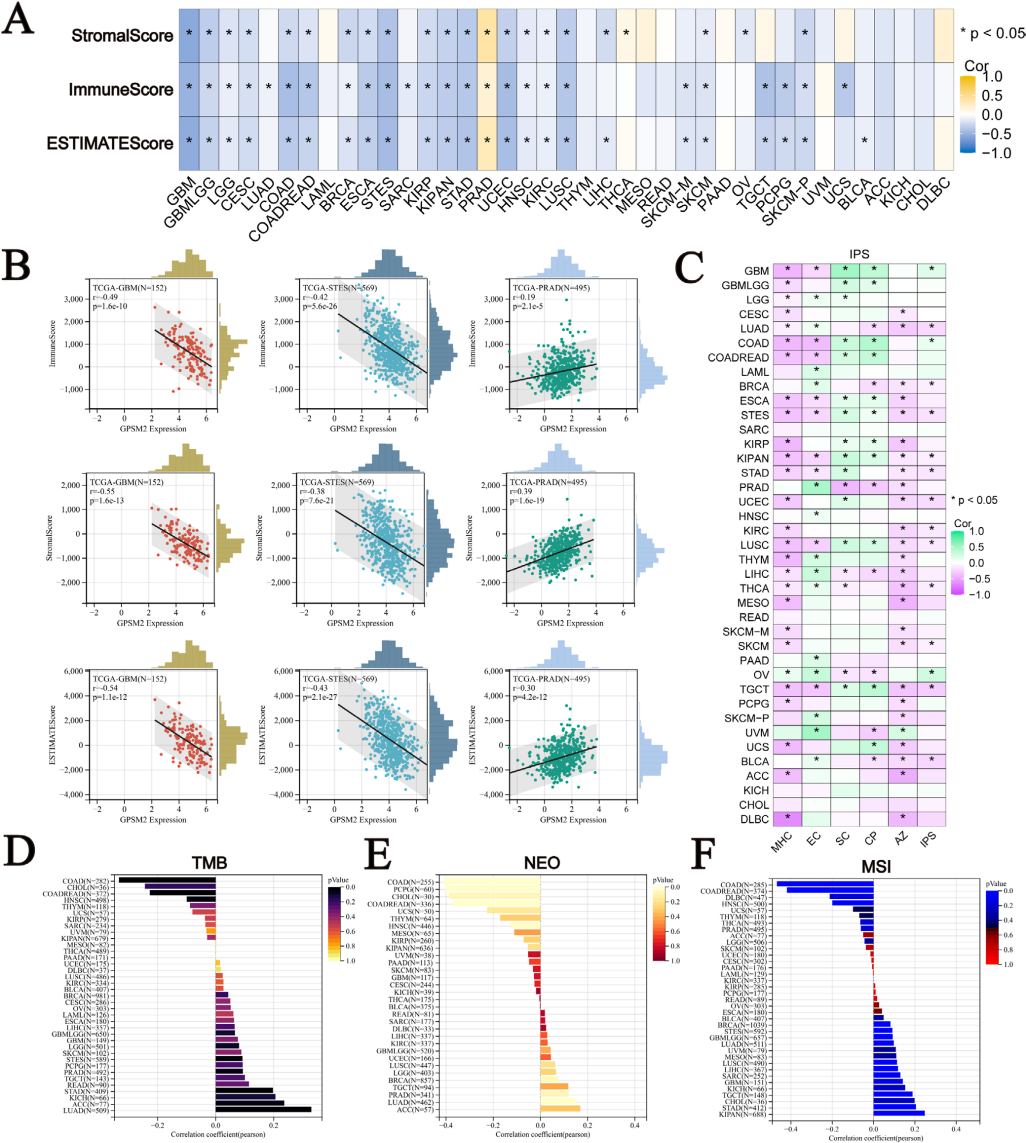
Correlation between tumor microenvironment characteristics and GPSM2 expression in pan‐cancer tissues. (A‐B) GPSM2 expression correlates with microenvironment score, stroma score, and ESTIMATE score. (C) Association of GPSM2 expression with immune subtypes: MHC, EC, SC, CP, AZ, and IPS. (D‐F) GPSM2 expression correlates with tumor mutational burden (TMB), neoantigen load (NEO), and microsatellite instability (MSI).

We calculated the correlation between GPSM2 and microenvironmental score, stromal score, and ESTIMATEScore, and found that most of the tumours were negatively correlated with the immune score and microenvironmental score, except for PRAD and THCA (Figure 7A). In GBM, stomach and oesophageal carcinoma (STES), and prostate adenocarcinoma (PRAD), the expression level of GPSM2 was strongly correlated with the immunity score (Figure 7B). We also analysed GPSM2 with MHC, EC, SC, CP, AZ, and IPS infiltration scores for each patient in each tumour type (Figure 7C). The results showed that GPSM2 expression was negatively correlated with IPS, MHC, and AZ in most tumours. TMB, MSI, and NEO are considered to be important in predicting tumour immunotherapy response. We observed a significant positive correlation of GPSM2 expression with TMB, NEO, and MSI in LUAD (Figure 7D–F). Whereas, the expression level of GPSM2 in COAD and uterine Carcinosarcoma (UCS) had a significant negative correlation with these three. These suggest that GPSM2 may play an important role in tumour immunity.

**FIGURE 8:**
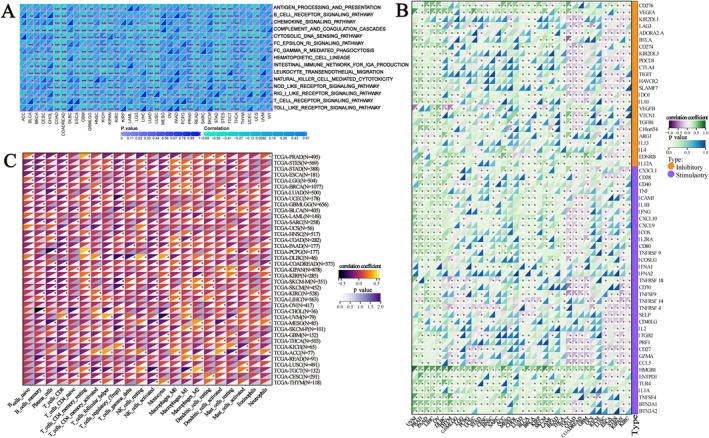
Mechanism of GPSM2 regulating the immune microenvironment.

We calculated the scores of immune‐related pathways and explored the relationship between GPSM2 and them. Among them, in COAD, lung squamous cell carcinoma (LUSC), UCS, and colon adenocarcinoma/rectum adenocarcinoma oesophageal carcinoma (COADREAD) these immune activities showed significant positive correlations with GPSM2 (Figure 8A). Meanwhile, GPSM2 was mostly positively correlated with immune checkpoint genes in UVM, PRAD, and PAAD, while it was mostly negatively correlated in kidney renal clear cell carcinoma (KIRC) and KIPAN (Figure 8B). In almost all tumours, GPSM2 showed a negative correlation with most of the immune cells (Figure 8C).

### 3.7 Relationship Between GPSM2 and Immune Cells in Spatial Transcriptome Data

**FIGURE 9:**
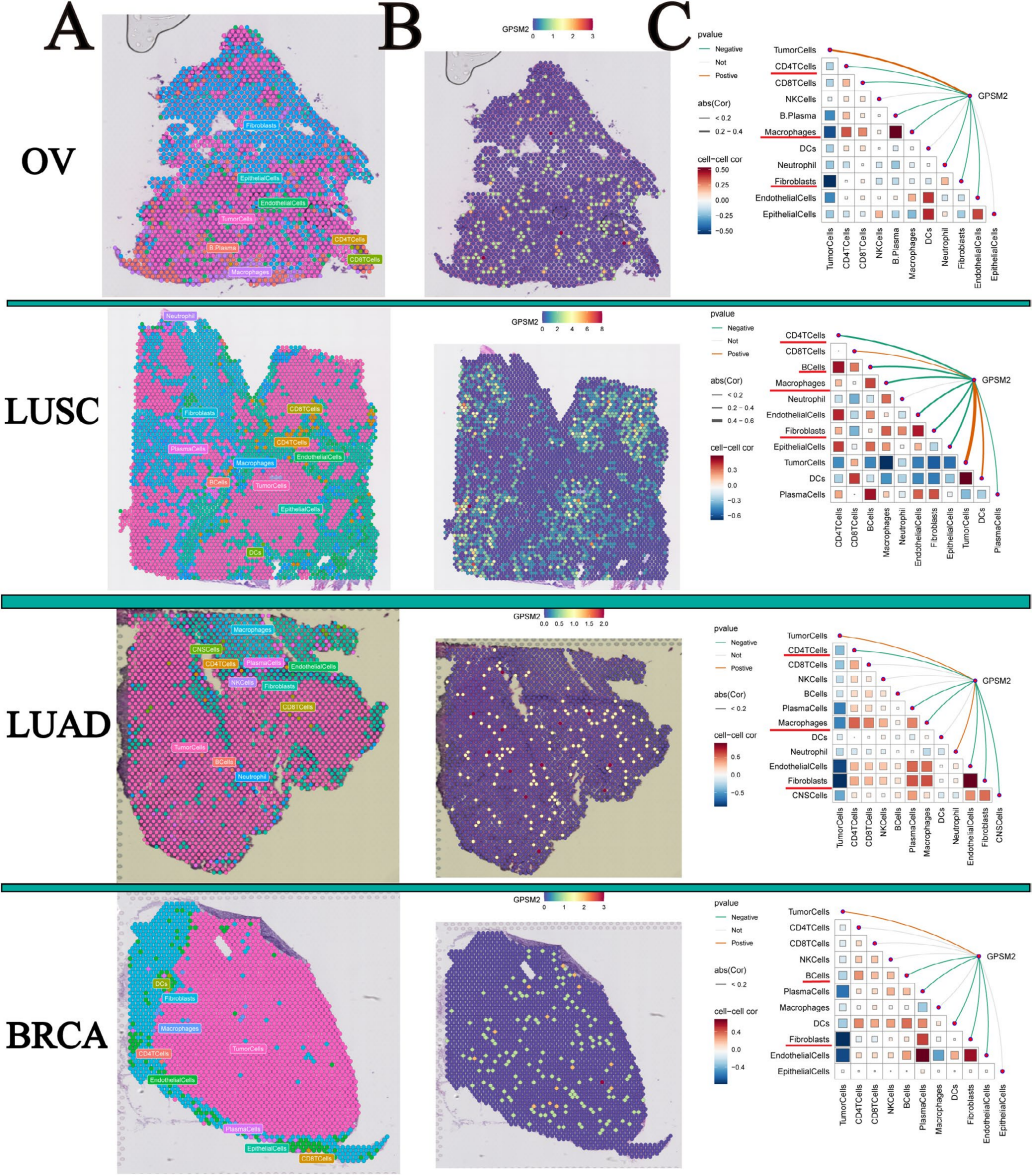
Spatial transcriptome reveals the spatial expression pattern of GPSM2.

Spatial transcriptome sequencing can simultaneously obtain spatial location information of cells and gene expression data, providing an important research tool to help understand tissue‐cell function and microenvironmental interactions. Therefore, we visualised the gene expression landscapes in each microregion using spatial transcriptome data. The higher the enrichment score, the darker was the colour, and the higher the content of this cell type in the spot. In addition, the analysis calculated the correlation between cell content and cell content, and between cell content and GPSM2 expression in all the spots (Figure 9A–C). Consistent with our results using transcriptomic data, GPSM2 showed a negative correlation with CD4T cells, fibroblasts, and macrophage cells in ovarian serous cystadenocarcinoma (OV), LUSC, LUAD, and BRCA. These results suggest that GPSM2 plays a role in the regulation of tumour immune cell infiltration.

### 3.8 Drug Sensitivity Analysis

Despite the increasing diversity of therapeutic approaches, resistance to chemotherapy and radiotherapy has caused certain drawbacks to traditional treatments [24]. The literature suggests that the development of drug resistance in tumours is associated with abnormal gene regulation [25, 26, 27].

**FIGURE 10:**
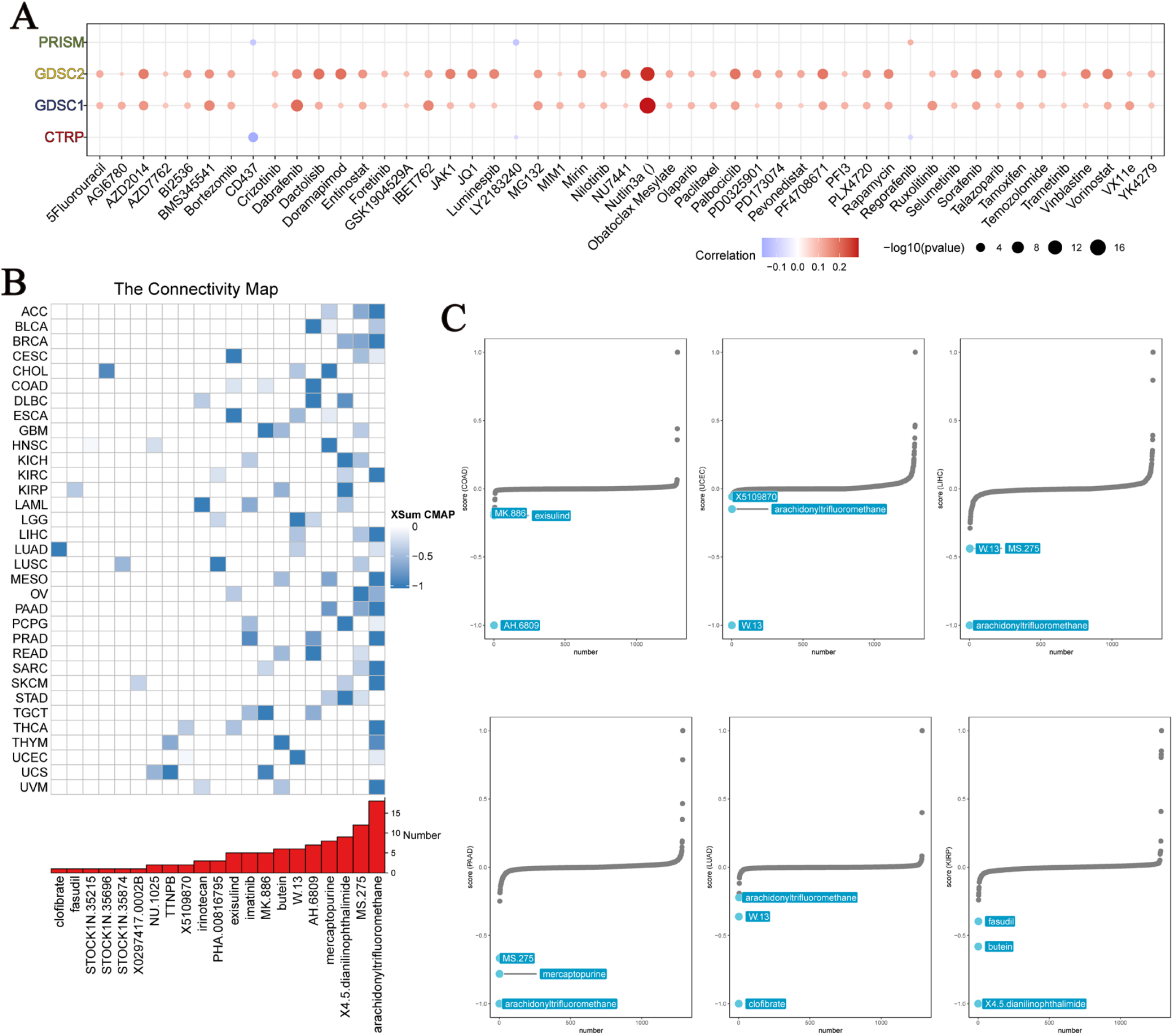
Prediction of GPSM2‐targeted drugs.

To determine whether GPSM2 plays a role in drug resistance, we analysed the association between GPSM2 and drug sensitivity. Information from four databases was used to perform a comprehensive analysis, and these data were obtained using experimental assays (Figure 10A). In both databases, GPSM2 showed a strong positive correlation with the IC50 value of Nutlin‐3a. Correlation coefficients close to 1, indicating a positive correlation between GPSM2 expression and IC50 values, usually imply that GPSM2 may play an important role in the drug response process or may be considered a potential biomarker for predicting drug sensitivity.

Connectivity map analysis was used to explore potential drugs targeting GPSM2. In more than 15 cancers, arachidonyl trifluoromethane likely reversed GPSM2 dysregulation and attenuated its oncogenic effects (Figure 10B). We also demonstrated that compounds targeting GPSM2 in six cancers had prognostic value (Figure 10C). These findings provide substantial support for our predicted effectiveness, although further studies are required to elucidate the underlying mechanisms.

### 3.9 Functional Enrichment Analysis and GSEA Analysis of GPSM2‐Related Genes

**FIGURE 11:**
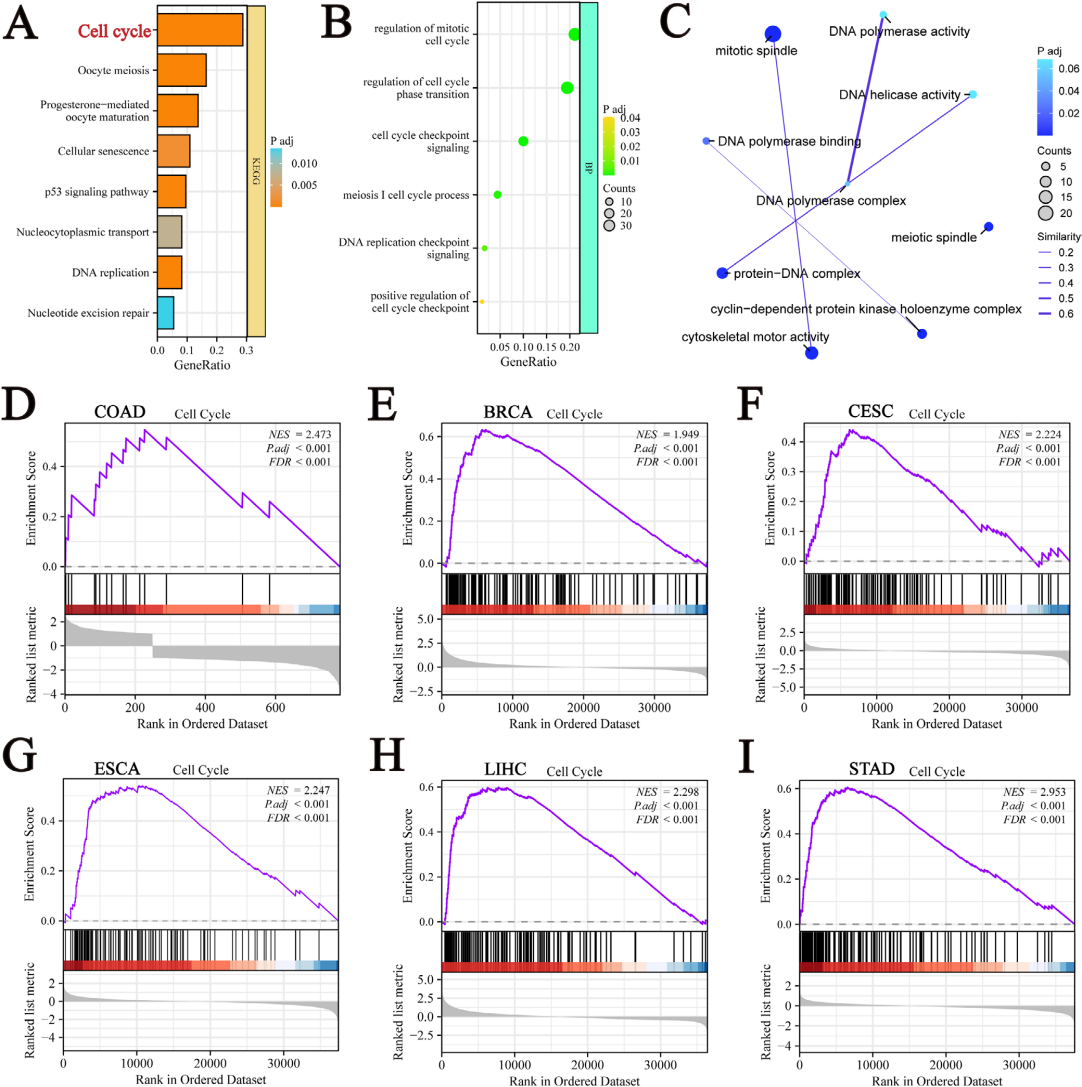
GPSM2 functional enrichment and signalling pathway.

To further elucidate the biological function of GPSM2 in pan‐cancer, the 200 genes most related to GPSM2 were obtained from the GEPIA2 database. Enrichment analysis showed that GPSM2 was closely associated with the cell cycle (Figure 11A–C). To further validate our findings, we applied GSEA based on GPSM2 differential expression analysis to elucidate the biological functions of GPSM2 in six tumours, including ACC (Figure 11D), KIRC (Figure 11E), kidney renal papillary cell carcinoma (KIRP) (Figure 11F), LUSC (Figure 11G), thyroid carcinoma (THCA) (Figure 11H), and pancreatic adenocarcinoma (PAAD) (Figure 11I). These results indicated that GPSM2 is closely related to the cell cycle.

### 3.10 Experimental Validation of GPSM2 in Colon Cancer Cells

**FIGURE 12:**
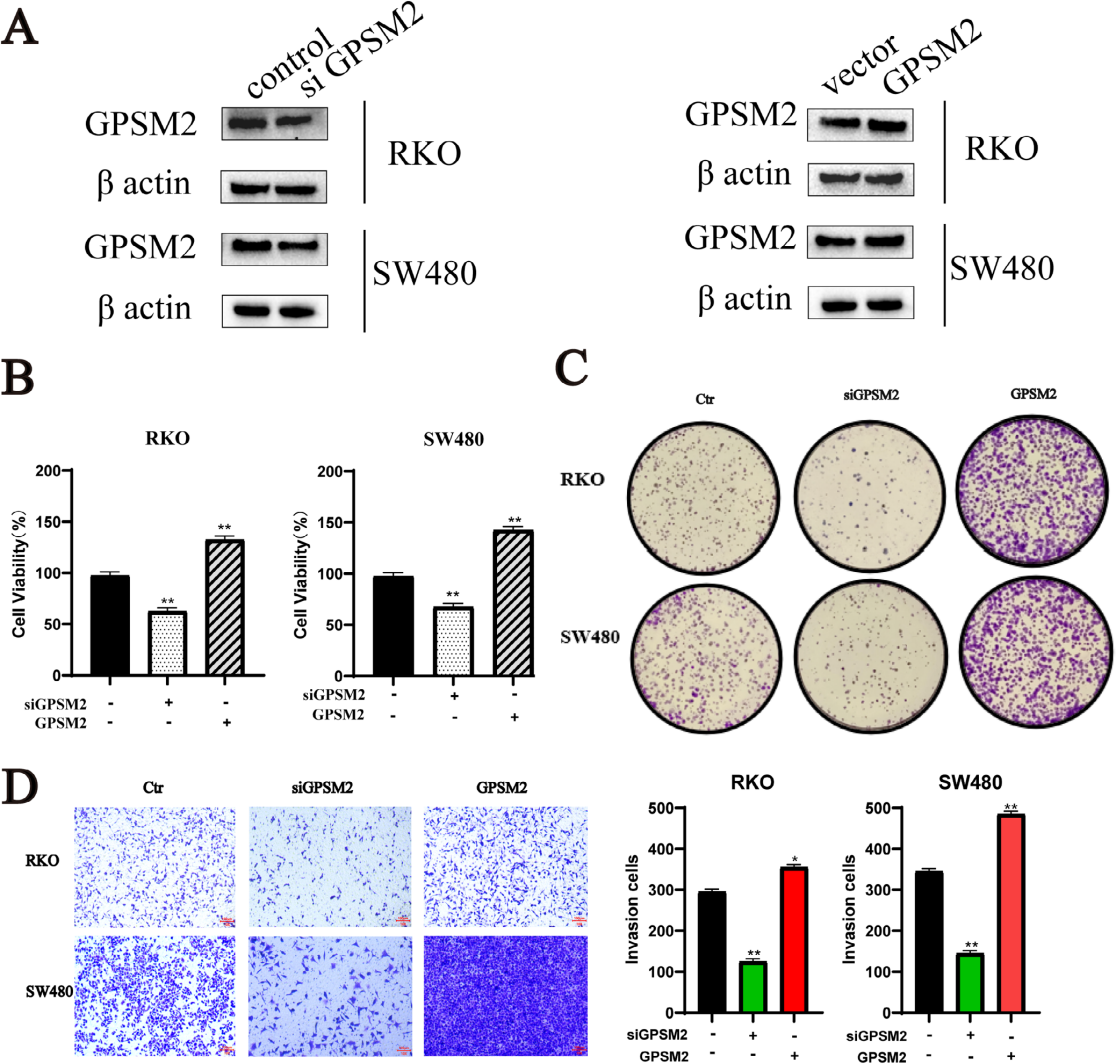
GPSM2 in vitro functional validation experiments.

**FIGURE 13:**
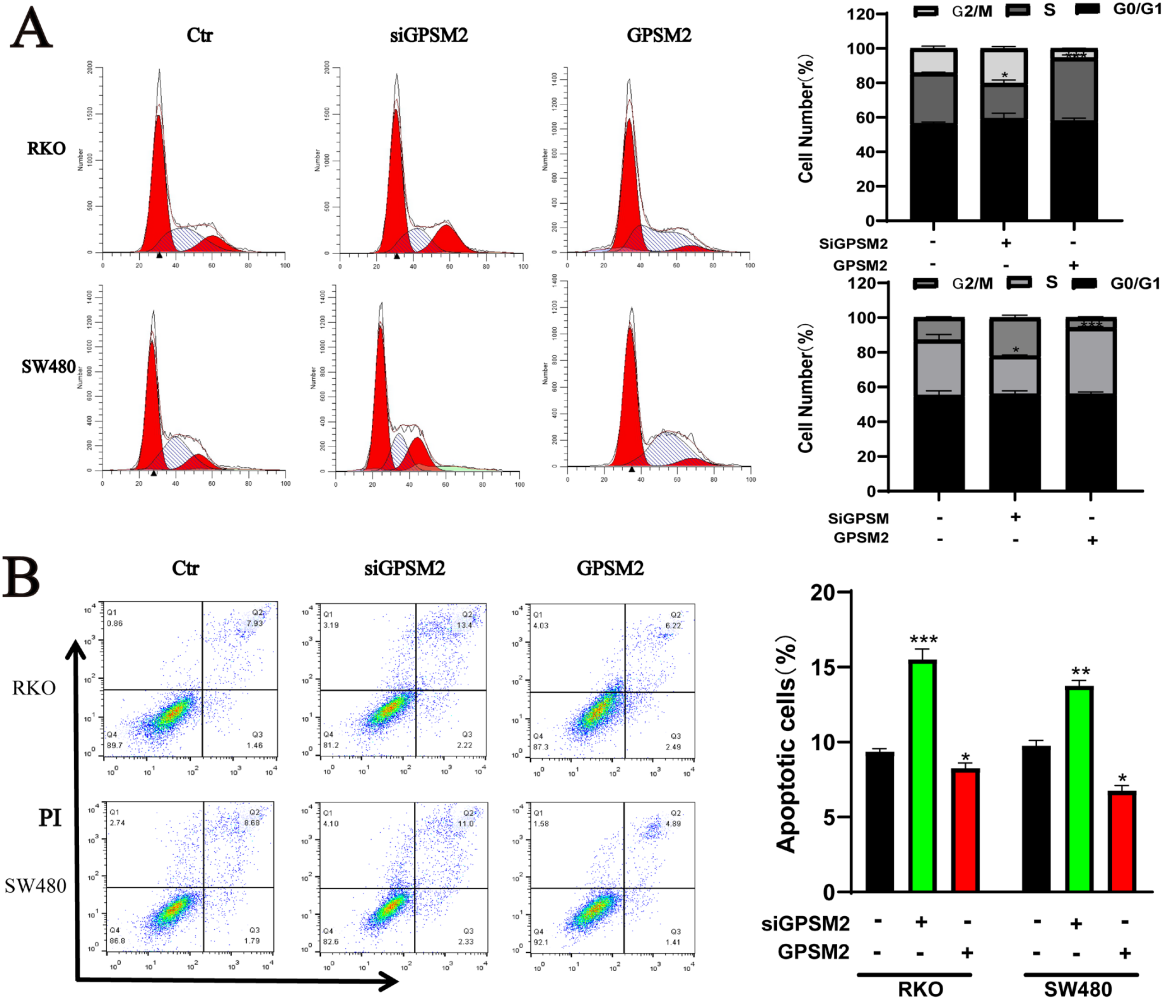
Regulation of cell cycle and apoptosis by GPSM2.

G‐protein signalling modulator 2 showed excellent diagnostic efficacy. We verified the effects of GPSM2 on the biological behaviours of RKO and SW480 cancer cells. Western blotting showed that the protein expression level of GPSM2 decreased and increased in the si‐GPSM2 and GPSM2 overexpression groups, respectively, when compared with the control group (Figure 12A). The CCK‐8 proliferation assay showed that compared with the control group, the si‐GPSM2 group had a reduced cell proliferation rate, whereas the cell proliferation rate was elevated in the GPSM2 overexpression group (Figure 12B). The si‐GPSM2 group showed a reduced cell migration rate, whereas it was increased in the GPSM2 overexpression group (Figure 12C). The cell invasion assay showed that the cell invasion rate was decreased in the si‐GPSM2 group and increased in the GPSM2 overexpression group (Figure 12D). The GPSM2 knockdown inhibited the proliferation of RKO and SW480 cells and induced apoptosis (Figure 13A,B). These results suggest that GPSM2 plays a key role in regulating cell cycle progression and apoptosis in colon cancer.

## 4 Discussion

We observed generalised and significant changes in the expression levels of GPSM2 in The Cancer Genome Atlas and GTEx pan‐cancer databases. Furthermore, GPSM2 has a prognostic value in a variety of cancers. Specifically, the expression level of GPSM2 closely correlated with prognostic indicators, including OS, DFS, DS, and PFI. In addition, GPSM2 has shown a good diagnostic value for a wide range of tumours. These analyses highlight GPSM2 as a promising biomarker for various cancers, highlighting its significant potential for clinical applications.

Three important forms of RNA methylation, m1A, m5C, and m6A, play key roles in regulating gene expression, RNA stability, and protein translation [28, 29]. Abnormalities of these modifications are closely related to the occurrence and development of many diseases, especially tumours [30]. We found that the expression of GPSM2 was positively correlated with m1A, m5C, and m6A genes, suggesting that GPSM2 may be involved in the regulatory process of RNA methylation modification. This regulation may be achieved by affecting the activity of related enzymes, RNA stability, or translation efficiency, which in turn affects the biological behaviours of tumour cells such as proliferation, differentiation, and apoptosis.

Tumour stemness is significantly correlated with immune response and heterogeneity in terms of prognosis [21, 31]. We found a significant positive correlation between GPSM2 expression and tumour stemness score in most malignant tumours. This may imply that the immune escape ability of tumour stem cells is stronger in tumours with high GPSM2 expression [32]. Given the important role of GPSM2 in the regulation of tumour stemness, it could be a potential target for the development of novel antitumour drugs [33]. Inhibiting the function or expression of GPSM2 may inhibit the growth and differentiation of tumour stem cells for tumour treatment [34].

The tumour microenvironment is a complex and evolving entity comprising primarily immune cells, stromal cells, blood vessels, and extracellular matrix [35]. The tumour microenvironment has been identified as playing a significant role in carcinogenesis, progression, and metastasis [36, 37, 38]. Therefore, we focused on the relationship between GPSM2 and the infiltration of immune cells. We found a significant negative correlation between GPSM2 expression and the infiltration of various immune cells, especially CD4T cells, Fibroblasts, and Macrophages, as calculated by various immunological algorithms. In addition, spatial transcriptome data supported this finding. The negative correlation between GPSM2 expression and immune cell infiltration indicates its potential role in regulating the tumour microenvironment by affecting immune cell distribution and function. Importantly, combining GPSM2 expression levels with immune cell infiltration can help us create more personalised tumour immunotherapy strategies. For example, in cancer patients with high GPSM2 expression and low immune cell infiltration, therapies targeting GPSM2 may be combined with immunotherapy to enhance treatment efficacy.

Tumour mutation burden, microsatellite instability, and neoantigens are significant indicators of the tumour microenvironment. Tumour mutation burden is frequently used as an indicator of the efficacy of immune checkpoint inhibitors [39]. Specifically, a consistently high tumour mutation burden tends to indicate better treatment outcomes [40, 41]. Microsatellite instability (MSI) is often used as an indicator to determine the prognosis of cancer treatment [39]. Neoantigens are considered excellent candidates for tumour immunotherapy because they are frequently expressed only in tumour cells and are associated with immunogenicity and tumour heterogeneity [42]. The positive correlation of GPSM2 with tumour mutation burden, neoantigens, and microsatellite instability in LUAD may imply that high GPSM2 expression in LUAD is associated with a more active immune response and higher tumour mutational load, which is usually a prerequisite for a good response to immunotherapies such as immune checkpoint inhibitors. This positive correlation may support GPSM2 as a biomarker for immunotherapeutic potential in LUAD. In addition, these observations provide clues for basic research, prompting investigators to further explore the specific mechanisms underlying the role of GPSM2 in tumorigenesis, development, and immune response.

Drug sensitivity data were obtained from four relevant databases. We analysed the correlation between GPSM2 expression and drug sensitivity from different perspectives. We demonstrated that GPSM2 is a potential target for tumour therapy by examining the link between GPSM2 expression and drug sensitivity. These results suggest that the occurrence of drug resistance in tumours may be related to abnormal GPSM2 expression. In addition, we found that compounds such as arachidonyl trifluoromethane and MS.275 could reverse GPSM2‐mediated carcinogenesis. These results also provide ideas for new strategies for tumour‐targeted therapy.

To understand the specific mechanism of action of GPSM2, we investigated the relationship between this gene and cancer‐related signalling pathways using large‐scale GSEA data. These results pave the way for future studies to investigate the molecular mechanisms underlying the role of GPSM2 in various tumours and for the development of therapeutic regimens. Specifically, we found that genes associated with GPSM2 were significantly correlated with the cell cycle, suggesting that GPSM2 influences tumour progression through its effects on the cell cycle. We confirmed our findings through in vitro experiments, which showed that silencing of GPSM2 inhibited cell proliferation, blocked the cell cycle, and induced apoptosis in the RKO and SW480 colon cancer cell lines. Furthermore, silencing of GPSM2 slowed the progression of colon cancer cells, which is consistent with previous reports on its role in non‐small cell lung, pancreatic, and breast cancer.

## 5 Conclusion

To our knowledge, this is the first study to report the role of GPSM2 in various cancer types. Our analysis revealed that GPSM2 is differentially expressed in various tumours compared with normal tissues. Furthermore, early monitoring of GPSM2 expression may aid in the diagnosis of several cancer types and predict poor prognosis. Additionally, we examined the relationship between GPSM2 expression and tumour stemness, RNA modifier genes, immune genes, immune infiltration, drug sensitivity, and cell cycle. These results demonstrate the importance of GPSM2 in tumour immunity as well as highlight its potential as a key candidate for antitumor immunotherapy. This study aimed to enhance our understanding of the role of GPSM2 in oncology, underscore its potential as a therapeutic target, and offer insights for further exploration of tumour pathogenesis.

## Author Contributions

**Chunjiao Yang:** methodology (lead), resources (lead), software (lead), writing – review and editing (lead). **Yuzhe Zhang:** data curation (lead), methodology (lead), visualization (lead), writing – original draft (lead). **Lirong Yan:** investigation (supporting), resources (supporting), software (supporting). **Aoran Liu:** resources (supporting), software (supporting). **Fang Li:** supervision (supporting), validation (supporting). **Yanke Li:** data curation (equal), formal analysis (equal), investigation (equal), project administration (lead). **Ye Zhang:** conceptualization (lead), funding acquisition (lead), supervision (lead), validation (lead).

## Ethics Statement

Ethical review and approval were waived for this study since no patient data were reported.

## Conflicts of Interest

The authors declare no conflicts of interest.

## Data Availability

All data generated or analysed during this study are included in this published article. The data used to support the findings of the present study are available from the corresponding author upon request.

## References

[1] H. Sung , J. Ferlay , R. L. Siegel , et al., “Global Cancer Statistics 2020: GLOBOCAN Estimates of Incidence and Mortality Worldwide for 36 Cancers in 185 Countries,” CA: A Cancer Journal for Clinicians 71, no. 3 (2021): 209–249. 33538338 10.3322/caac.21660

[2] S. Srivastava , E. J. Koay , A. D. Borowsky , et al., “Cancer Overdiagnosis: A Biological Challenge and Clinical Dilemma,” Nature Reviews. Cancer 19, no. 6 (2019): 349–358. 31024081 10.1038/s41568-019-0142-8 PMC8819710

[3] M. F. Sanmamed and L. Chen , “A Paradigm Shift in Cancer Immunotherapy: From Enhancement to Normalization,” Cell 175, no. 2 (2018): 313–326. 30290139 10.1016/j.cell.2018.09.035 PMC6538253

[4] B. Ricciuti , X. Wang , J. V. Alessi , et al., “Association of High Tumor Mutation Burden in Non‐Small Cell Lung Cancers With Increased Immune Infiltration and Improved Clinical Outcomes of PD‐L1 Blockade Across PD‐L1 Expression Levels,” JAMA Oncology 8, no. 8 (2022): 1160–1168. 35708671 10.1001/jamaoncol.2022.1981 PMC9204620

[5] A. G. Waks and E. P. Winer , “Breast Cancer Treatment: A Review,” JAMA 321, no. 3 (2019): 288–300. 30667505 10.1001/jama.2018.19323

[6] F. Ju , Y. Luo , C. Lin , et al., “Oncolytic Virus Expressing PD‐1 Inhibitors Activates a Collaborative Intratumoral Immune Response to Control Tumor and Synergizes With CTLA‐4 or TIM‐3 Blockade,” Journal for Immunotherapy of Cancer 10, no. 6 (2022): e004762. 35688558 10.1136/jitc-2022-004762 PMC9189843

[7] L. Yan , S. Shen , A. Wang , et al., “Comprehensive Pan‐Cancer Analysis of Heat Shock Protein 110, 90, 70, and 60 Families,” Frontiers in Molecular Biosciences 8 (2021): 726244, 10.3389/fmolb.2021.726244. 34712697 PMC8546173

[8] S. Xu , Y. Zheng , M. Ye , et al., “Comprehensive Pan‐Cancer Analysis Reveals EPHB2 Is a Novel Predictive Biomarker for Prognosis and Immunotherapy Response,” BMC Cancer 24, no. 1 (2024): 1064, 10.1186/s12885-024-12843-0. 39198775 PMC11351591

[9] Z. Zhu , W. Zhang , S. Huo , T. Huang , X. Cao , and Y. Zhang , “TUBB, a Robust Biomarker With Satisfying Abilities in Diagnosis, Prognosis, and Immune Regulation via a Comprehensive Pan‐Cancer Analysis,” Frontiers in Molecular Biosciences 11 (2024): 1365655, 10.3389/fmolb.2024.1365655. 38756529 PMC11096532

[10] J. Lin , P. Liu , K. Sun , et al., “Comprehensive Analysis of KLF Family Reveals KLF6 as a Promising Prognostic and Immune Biomarker in Pancreatic Ductal Adenocarcinoma,” Cancer Cell International 24, no. 1 (2024): 177. 38773440 10.1186/s12935-024-03369-3 PMC11106939

[11] D. Liu , J. Peng , J. Xie , and Y. Xie , “Comprehensive Analysis of the Function of Helicobacter‐Associated Ferroptosis Gene YWHAE in Gastric Cancer Through Multi‐Omics Integration, Molecular Docking, and Machine Learning,” Apoptosis 29, no. 3 (2024): 439–456, 10.1007/s10495-023-01916-3. 38001345

[12] H. Wu , Q. Geng , W. Shi , and C. Qiu , “Comprehensive Pan‐Cancer Analysis Reveals CCDC58 as a Carcinogenic Factor Related to Immune Infiltration,” Apoptosis 29, no. 3 (2024): 536–555, 10.1007/s10495-023-01919-0. 38066393

[13] P. Samadi , M. Shahnazari , A. Shekari , F. Maghool , and A. Jalali , “A Pan‐Cancer Analysis Indicates Long Noncoding RNA HAND2‐AS1 AS a Potential Prognostic, Immunomodulatory and Therapeutic Biomarker in Various Cancers Including Colorectal Adenocarcinoma,” Cancer Cell International 23, no. 1 (2023): 307, 10.1186/s12935-023-03163-7. 38042769 PMC10693120

[14] D. Doherty , A. E. Chudley , G. Coghlan , et al., “GPSM2 Mutations Cause the Brain Malformations and Hearing Loss in Chudley‐McCullough Syndrome,” American Journal of Human Genetics 90, no. 6 (2012): 1088–1093. 22578326 10.1016/j.ajhg.2012.04.008 PMC3370271

[15] C. Fukukawa , K. Ueda , T. Nishidate , T. Katagiri , and Y. Nakamura , “Critical Roles of LGN/GPSM2 Phosphorylation by PBK/TOPK in Cell Division of Breast Cancer Cells,” Genes, Chromosomes & Cancer 49, no. 10 (2010): 861–872. 20589935 10.1002/gcc.20795

[16] M. Deng , B. Liu , Z. Zhang , et al., “Knockdown of G‐Protein‐Signaling Modulator 2 Promotes Metastasis of Non‐Small‐Cell Lung Cancer by Inducing the Expression of Snail,” Cancer Science 111, no. 9 (2020): 3210–3221, 10.1111/cas.14519. 32519357 PMC7469834

[17] P. J. Thul , L. Åkesson , M. Wiking , et al., “A Subcellular Map of the Human Proteome,” Science 356, no. 6340 (2017): eaal3321. 28495876 10.1126/science.aal3321

[18] W. Shen , Z. Song , X. Zhong , et al., “Sangerbox: A Comprehensive, Interaction‐Friendly Clinical Bioinformatics Analysis Platform,” iMeta 1 (2022): e36, 10.1002/imt2.36. 38868713 PMC10989974

[19] D. Zeng , Z. Ye , and R. Shen , “IOBR: Multi‐Omics Immuno‐Oncology Biological Research to Decode Tumor Microenvironment and Signatures,” Frontiers in Immunology 12 (2021): 687975, https://pubmed.ncbi.nlm.nih.gov/34276676/. 34276676 10.3389/fimmu.2021.687975 PMC8283787

[20] P. Charoentong , F. Finotello , M. Angelova , et al., “Pan‐Cancer Immunogenomic Analyses Reveal Genotype‐Immunophenotype Relationships and Predictors of Response to Checkpoint Blockade,” Cell Reports 18, no. 1 (2017): 248–262. 28052254 10.1016/j.celrep.2016.12.019

[21] T. M. Malta , A. Sokolov , A. J. Gentles , et al., “Machine Learning Identifies Stemness Features Associated With Oncogenic Dedifferentiation,” Cell 173, no. 2 (2018): 338–354. 29625051 10.1016/j.cell.2018.03.034 PMC5902191

[22] Z. Xun , X. Ding , Y. Zhang , et al., “Reconstruction of the Tumor Spatial Microenvironment Along the Malignant‐Boundary‐Nonmalignant Axis,” Nature Communications 14, no. 1 (2023): 933. 10.1038/s41467-023-36560-7 PMC9941488 36806082

[23] J. Shi , X. Wei , Z. Xun , et al., “The Web‐Based Portal SpatialTME Integrates Histological Images With Single‐Cell and Spatial Transcriptomics to Explore the Tumor Microenvironment,” Cancer Research 84, no. 8 (2024): 1210–1220. 38315776 10.1158/0008-5472.CAN-23-2650

[24] Y. Sun , “Translational Horizons in the Tumor Microenvironment: Harnessing Breakthroughs and Targeting Cures,” Medicinal Research Reviews 35, no. 2 (2015): 408–436. 25588753 10.1002/med.21338 PMC4374701

[25] A. F. Aissa , A. B. M. M. K. Islam , M. M. Ariss , et al., “Single‐Cell Transcriptional Changes Associated With Drug Tolerance and Response to Combination Therapies in Cancer,” Nature Communications 12, no. 1 (2021): 1628. 10.1038/s41467-021-21884-z PMC7955121 33712615

[26] K. Cho , X. Wang , S. Nie , Z. G. Chen , and D. M. Shin , “Therapeutic Nanoparticles for Drug Delivery in Cancer,” Clinical Cancer Research 14, no. 5 (2008): 1310–1316. 18316549 10.1158/1078-0432.CCR-07-1441

[27] K. Han , S. Chen , W.‐H. Chen , et al., “Synergistic Gene and Drug Tumor Therapy Using a Chimeric Peptide,” Biomaterials 34, no. 19 (2013): 4680–4689. 23537665 10.1016/j.biomaterials.2013.03.010

[28] T. Pan , “Modifications and Functional Genomics of Human Transfer RNA,” Cell Research 28, no. 4 (2018): 395–404. 29463900 10.1038/s41422-018-0013-y PMC5939049

[29] W.‐W. Liu , S.‐Q. Zheng , T. Li , et al., “RNA Modifications in Cellular Metabolism: Implications for Metabolism‐Targeted Therapy and Immunotherapy,” Signal Transduction and Targeted Therapy 9, no. 1 (2024): 70. 38531882 10.1038/s41392-024-01777-5 PMC10966055

[30] Y. An and H. Duan , “The Role of m6A RNA Methylation in Cancer Metabolism,” Molecular Cancer 21, no. 1 (2022): 14. 35022030 10.1186/s12943-022-01500-4 PMC8753874

[31] A. Miranda , P. T. Hamilton , A. W. Zhang , et al., “Cancer Stemness, Intratumoral Heterogeneity, and Immune Response Across Cancers,” Proceedings of the National Academy of Sciences of the United States of America 116, no. 18 (2019): 9020–9029. 30996127 10.1073/pnas.1818210116 PMC6500180

[32] T. Huang , X. Song , D. Xu , et al., “Stem Cell Programs in Cancer Initiation, Progression, and Therapy Resistance,” Theranostics 10, no. 19 (2020): 8721–8743. 32754274 10.7150/thno.41648 PMC7392012

[33] J. A. Clara , C. Monge , Y. Yang , and N. Takebe , “Targeting Signalling Pathways and the Immune Microenvironment of Cancer Stem Cells ‐ a Clinical Update,” Nature Reviews. Clinical Oncology 17, no. 4 (2020): 204–232. 10.1038/s41571-019-0293-2 31792354

[34] A. Agliano , A. Calvo , and C. Box , “The Challenge of Targeting Cancer Stem Cells to Halt Metastasis,” Seminars in Cancer Biology 44 (2017): 25–42. 28323021 10.1016/j.semcancer.2017.03.003

[35] M. Binnewies , E. W. Roberts , K. Kersten , et al., “Understanding the Tumor Immune Microenvironment (TIME) for Effective Therapy,” Nature Medicine 24, no. 5 (2018): 541–550. 10.1038/s41591-018-0014-x PMC5998822 29686425

[36] J. Zhang , H. Tavakoli , L. Ma , X. Li , L. Han , and X. Li , “Immunotherapy Discovery on Tumor Organoid‐On‐a‐Chip Platforms That Recapitulate the Tumor Microenvironment,” Advanced Drug Delivery Reviews 187 (2022): 114365. 35667465 10.1016/j.addr.2022.114365

[37] D. Kreatsoulas , C. Bolyard , B. X. Wu , H. Cam , P. Giglio , and Z. Li , “Translational Landscape of Glioblastoma Immunotherapy for Physicians: Guiding Clinical Practice With Basic Scientific Evidence,” Journal of Hematology & Oncology 15, no. 1 (2022): 80. 35690784 10.1186/s13045-022-01298-0 PMC9188021

[38] T. Eisemann and R. J. Wechsler‐Reya , “Coming in From the Cold: Overcoming the Hostile Immune Microenvironment of Medulloblastoma,” Genes & Development 36, no. 9–10 (2022): 514–532. 35680424 10.1101/gad.349538.122 PMC9186392

[39] P. S. Hegde and D. S. Chen , “Top 10 Challenges in Cancer Immunotherapy,” Immunity 52, no. 1 (2020): 17–35. 31940268 10.1016/j.immuni.2019.12.011

[40] J. Y. Kim , A. Kronbichler , M. Eisenhut , et al., “Tumor Mutational Burden and Efficacy of Immune Checkpoint Inhibitors: A Systematic Review and Meta‐Analysis,” Cancers 11, no. 11 (2019): 1798. 31731749 10.3390/cancers11111798 PMC6895916

[41] D. M. Merino , L. M. McShane , D. Fabrizio , et al., “Establishing Guidelines to Harmonize Tumor Mutational Burden (TMB): In Silico Assessment of Variation in TMB Quantification Across Diagnostic Platforms: Phase I of the Friends of Cancer Research TMB Harmonization Project,” Journal for Immunotherapy of Cancer 8, no. 1 (2020): e000147. 32217756 10.1136/jitc-2019-000147 PMC7174078

[42] M. Peng , Y. Mo , Y. Wang , et al., “Neoantigen Vaccine: An Emerging Tumor Immunotherapy,” Molecular Cancer 18, no. 1 (2019): 128. 31443694 10.1186/s12943-019-1055-6 PMC6708248

